# Consumption of Dietary Supplements among Working-Age Residents of Lithuania in the Period from 2021 to 2023

**DOI:** 10.3390/medicina60040669

**Published:** 2024-04-19

**Authors:** Rokas Arlauskas, Donatas Austys, Valerij Dobrovolskij, Rimantas Stukas

**Affiliations:** Department of Public Health, Institute of Health Sciences, Faculty of Medicine, Vilnius University, M. K. Čiurlionio 21/27, 03101 Vilnius, Lithuania; donatas.austys@mf.vu.lt (D.A.); valerij.dobrovolskij@mf.vu.lt (V.D.); rimantas.stukas@mf.vu.lt (R.S.)

**Keywords:** dietary supplements, adults, working-age, Lithuania, health assessment

## Abstract

*Background and Objectives:* The aim of this study was to assess the consumption of dietary supplements (DS) among working-age residents of Lithuania from 2021 to 2023 with respect to social and demographic factors and an assessment of personal health. *Materials and Methods:* Using stratified sampling techniques, this study included three samples of working-age residents (1600 each year, 4800 total). Three surveys were conducted, the distribution of the respondents between groups was compared using the χ^2^ test. *Results:* The consumption of DS significantly differed each year and accounted for 78.1%, 71.6%, and 72.7% of the respondents, respectively (*p* < 0.05). In 2022, the prevalence of the consumption of DS was lower in the majority of social and demographic groups (*p* < 0.05). In 2023, it was higher among females, younger residents, and those from larger families, who suffered from COVID-19 (*p* < 0.05). Despite similar changes found in the consumption of DS among those who negatively assessed their health, this group showed more prevalent consumption of DS among residents with non-university education, unemployed respondents, and those with lower income (*p* < 0.05). *Conclusions:* Despite a significantly lower prevalence in the consumption of DS in 2022, it was higher again in 2023. The assessment of personal health shows different habits in the consumption of DS.

## 1. Introduction

When nutrition does not meet recommendations, dietary supplements may help correct micronutrient deficiency and maintain adequate intake [1]. On the other hand, overconsumption of dietary supplements should be avoided because of possible adverse health effects [2], especially those triggered by food supplements purchased illegally [3]. However, researchers bring to light a lack of awareness of these products observed among their consumers [3,4]. Major determinants of the consumption of dietary supplements include not only personal factors such as sociodemographic characteristics, older age, perceived benefits of dietary supplements, the history of illness, physiological conditions, and lifestyle factors, but also socio-economic factors like subjective norms, the price of food, and commercial considerations of the sectors involved in the production and sale of dietary supplements [2,5]. Attention should be drawn to the fact that dietary supplements are most commonly taken by people with no clinical signs or symptoms of deficiency [1], especially those with an interest in physical performance. Moreover, consumers of dietary supplements tend to have a better overall diet quality with their nutrient intake from foods that most commonly meet the recommended intake levels [1,6,7].

Multiple studies have shown that the COVID-19 pandemic had a significant impact on the prevalence of the consumption of dietary supplements [8,9,10,11]. Even though some of them revealed an increase in consumption [8,9,10], there were studies highlighting a decrease in the prevalence of the consumption of dietary supplements [11]. In addition to this, it was found that during the COVID-19 pandemic, the perceptions of dietary supplements changed. If, before the pandemic, dietary supplements were associated with healthcare and life cycle-related topics, for example, pregnancy, after the COVID-19 pandemic, consumer interests have shifted to disease prevention [12].

Despite the fact that sex, age, education, place of residence, marital status, number of family members, presence of children in the family, employment, income, COVID-19 cases in a family, and food selection criteria were analyzed as determinants of the nutritional habits and consumption of dietary supplements in previous studies, there is a lack of country-representative studies on the ongoing trends in the consumption of dietary supplements after the COVID-19 pandemic. There is an insufficient level of research carried on DS consumption among diverse social and demographic groups, especially among those with a negative assessment of personal health. After taking into account the inequalities in the consumption of dietary supplements and the occurrence of unequal changes in consumption during the COVID-19 pandemic [8], the aim of this study was to assess the consumption of dietary supplements among working-age residents of Lithuania in the period from 2021 to 2023 with respect to social and demographic factors and the assessment of personal health.

## 2. Materials and Methods

### 2.1. Procedure of Data Collection

The data for this study were collected after conducting three independent cross-sectional surveys in October and November of 2021, in October and November of 2022, and in October and November of 2023. A representative sample of adults aged 18 to 64 was formed each year. The multistage stratified probabilistic sampling method was used to select participants for this study. It ensured an equal probability for every household in the country to be surveyed, and, according to target criteria (sex, age, municipality, education, income, employment, marital status), the sample represented the general population of working-aged citizens of Lithuania. Data was collected by conducting an internet-based survey. Random samples of citizens were formed according to the Registry of Residents of Lithuania. Every selected resident received an invitation to participate in this study with a link to the anonymous questionnaire by email. The participants of this study filled out the questionnaire by themselves at a time that was convenient to them. It was possible to fill out the questionnaire only once. Only working-aged citizens of Lithuania were included in this study. This study did not include refugees and other people without Lithuanian citizenship.

Each of the samples independently included 1600 residents. The design of this study was not longitudinal. No data about the inclusion of the respondents in more than one sample were collected. In total, the answers of 4800 respondents are analyzed in this paper.

The comparison of the consumption of dietary supplements before the COVID-19 pandemic with the data collected during the first survey in 2021 has already been published [8]. The current paper focuses on the post-pandemic period and the analysis of the prevalence of the consumption of dietary supplements with respect to the subjective assessment of personal health, which had not been covered in our previous paper.

This study was reviewed by the Vilnius Regional Ethics Committee for Biomedical Research.

### 2.2. Description of the Questionnaire

Each of the three surveys was carried out using the same questionnaire with a minimal adaptation for the post-pandemic period. An anonymous questionnaire included questions about the social and demographic characteristics of the respondents, the COVID-19 cases in respondents’ families or among friends, their subjective assessment of personal health, nutrition, consumption of food supplements and physical activity. The questionnaire was formed on the basis of the previously used questionnaire about nutrition and consumption of food supplements [13]. In this paper, we present the analysis of the questions included in the questionnaire (Table 1).

Two of the questions regarding the respondents’ age and place of residence were open-ended. To achieve an unambiguous interpretation of the results, we transformed them into a binary format. Respondents were asked to identify the municipality they live in. Respondents from 5 municipalities with the largest number of residents were assigned to the “City” group, while the remaining respondents were attributed to the “Towns and villages” group. The age was categorized by median to the range up to 41-year-olds and from 42-year-olds. All other questions were closed. Respondents with primary or secondary education and high school graduates were assigned to the “Non-university education” group. Respondents with unfinished or finished university studies were assigned to the “University education” group. In terms of employment status, the “Employed” and “Unemployed” groups were created. Heads of companies or departments, office workers, civil servants, service sector employees, sellers, workers, and farmers were assigned to the “Employed” group. Retirees, housewives, persons on parental leave, non-employed persons and students were categorized into the “Unemployed” group. The variable representing an income per member of a family was transformed into a binary format with “Higher income” and “Lower income” categories. With respect to a salary increase, the cut-off point for those groups was 350 EUR in 2021, 400 EUR in 2022, and 470 EUR in 2023. In addition to this, more binary variables were created, such as the number of family members, marital status, and children under 18 years old. The categorization of the rest of the questionnaire is presented in Table 2.

### 2.3. Statistical Analysis

The normality of the variable representing the age of respondents in general samples was tested using the Kolmogorov–Smirnov test with Lilliefors significance correction. This test showed non-normal distributions. Therefore, medians with an interquartile range (Q1–Q3) were presented for this variable. The Pearson’s chi-squared test (χ^2^) was used to determine whether there was a statistically significant difference between the expected frequencies and the observed frequencies in one or more of the categories. Differences were considered statistically significant when the *p*-value was lower than 0.05.

## 3. Results

In total, this study included 4800 respondents: 1600 in 2021, 1600 in 2022 and 1600 in 2023. The median age in the samples collected in 2021 and 2022 was 42 (29–54) years, while in the sample of 2023 it was 43 (30–54) years. The majority of the respondents were employed, married (or with partners), from small towns or villages, with university education, and without children under 18 years old. The samples were similar in terms of sex, age, education, type of place of residence, children under 18 years old, employment status, income, and the subjective assessment of personal health (*p* > 0.05). The sample collected in 2022 compared to that in 2021 included relatively more single respondents and those who selected foods following other than strengthening health criteria (*p* < 0.05). In comparison to the 2022 sample, the 2023 sample included relatively more married (or with partners) respondents and those who selected foods with the aim of strengthening of their health (*p* < 0.05). The distribution of the respondents by social and demographic factors is presented in Table 2.

In 2021, the consumption of dietary supplements was prevalent among 1240 (78.1%) respondents. In 2022, the prevalence of the consumption of dietary supplements significantly lowered and accounted for 1131 (71.6%) subjects (*p* < 0.001). In 2023, compared to 2022, a higher prevalence of the consumption of dietary supplements was observed: 1163 (72.7%) respondents indicated the consumption of dietary supplements (*p* = 0.044). Nevertheless, the distribution of the respondents by the majority of purposes for the consumption of dietary supplements remained similar (*p* > 0.05). The consumption of dietary supplements with the aim of protection against the COVID-19 infection in 2023 was significantly lower than in the previous years (*p* = 0.004) (Table 3).

The comparison of the prevalence of the consumption of dietary supplements between the three samples revealed the differences within all social and demographic groups (*p* < 0.05) except for the respondents with children under 18 years old, unemployed respondents, those with lower income, those who indicated health strengthening as the main criterion for the selection of foods, and those who suffered from the asymptomatic or mild form of COVID-19 (*p* > 0.05). In 2022, the lower prevalence of the consumption of dietary supplements was observed in almost all social and demographic groups: among males, females, employed, younger and older residents of Lithuania, those with and without university education, those from big and small municipalities, those with and without a partner, from families of at least two members, without children, with lower and higher income, those who selected their foods for other than health strengthening criteria, those with and without COVID-19 cases in their families, those who suffered from a severe form of COVID-19, and those who assessed their health positively or negatively (*p* < 0.05). In terms of the prevalence of the consumption of dietary supplements, the samples of 2021 and 2022 did not differ among the respondents of one-person families, those with children under 18 years old, those who suffered from the asymptomatic or mild form of COVID-19, and those who indicated health strengthening as the main criteria for the selection of foods (*p* > 0.05). In 2023, no difference in the prevalence of the consumption of dietary supplements was found within all social and demographic groups (*p* > 0.05), except for a higher prevalence among females, younger residents of Lithuania, those from families with two or more members, those who suffered from COVID-19, and those who suffered from a severe form of COVID-19 (*p* < 0.05) (Table 4).

In at least two of the samples, a higher prevalence of consumption of dietary supplements was observed among females, those with university education, those from larger municipalities, employed respondents, those with higher income, those who suffered from COVID-19, and those who indicated health strengthening as the main criteria for the selection of foods (*p* < 0.05) (Table 4).

The comparison of the prevalence of a negative assessment of personal health between the three samples revealed no difference (*p* > 0.05) within all the social and demographic groups except among females, residents from smaller municipalities, adults with lower income, and those who suffered from the asymptomatic or mild form of COVID-19 (*p* < 0.05). In 2022, a higher prevalence of a negative assessment of personal health was observed among employed respondents and those with lower income (*p* < 0.05). A lower prevalence was observed among those who suffered from the asymptomatic or mild form of COVID-19. In 2023, a higher prevalence of a negative assessment of personal health was observed among females and those who suffered from the asymptomatic or mild form of COVID-19 (*p* < 0.05) (Table 5).

In at least two of the samples, a higher prevalence of the negative assessment of personal health was observed among older, unemployed respondents, those with non-university education, without children, with lower income, and those who indicated other than health strengthening as the main criteria for selecting foods (*p* < 0.05) (Table 5).

After comparing the prevalence of the consumption of dietary supplements among those who negatively assessed their personal health between the three samples, the differences were observed among males, older, employed respondents, those with non-university education, without children, those from families consisting of two or more members, those who indicated health strengthening and other criteria as the main criteria when selecting foods, and those with various forms of COVID-19 (*p* < 0.05). Among those who negatively assessed their personal health in 2022, a significantly lower prevalence of the consumption of dietary supplements was observed among males, older, employed respondents, those with non-university education, residents from cities and smaller municipalities, those with and without a partner, those from families with two or more members, those with and without children under 18 years old, those who indicated other than health strengthening as the main criteria for the selection of foods, and those who suffered from a severe form of COVID-19 (*p* < 0.05). Among those who negatively assessed their personal health in 2023, no difference in the prevalence of the consumption of dietary supplements was found compared to the previous year (*p* > 0.05), except for a higher prevalence among those who suffered from a severe form of COVID-19 and those who indicated strengthening of health as the main criterion for the selection of foods (*p* < 0.05) (Table 6).

In at least two of the samples, among the respondents who assessed their health negatively, a higher prevalence of the consumption of dietary supplements was observed among older respondents, those with non-university education, unemployed respondents, those with lower income, those who suffered from a severe form of COVID-19, and those who indicated other than health strengthening as the main criteria for the selection of foods (*p* < 0.05) (Table 6).

In all three samples, the top six reasons for the consumption of dietary supplements among the respondents who assessed their health negatively were strengthening the immune system, the overall strengthening of the body, strengthening the cardiovascular system, strengthening the bones and joints, boosting the nervous system, and improving digestion. Except for the lower prevalence of the consumption of dietary supplements with the aim to strengthen the cardiovascular system in 2022 (*p* < 0.05), the distribution pattern of the respondents who assessed their health negatively by purpose for the consumption of dietary supplements was similar in all three samples (*p* > 0.05) (Table 7).

In all three samples, the consumption of dietary supplements for better digestion was more prevalent among those who assessed their health negatively in comparison to those who assessed their health positively (*p* < 0.05). This trend was also observed with dietary supplements for the regulation of sleep and boosting the nervous system in 2021 and 2022, as well as for strengthening the cardiovascular system in 2021 (*p* < 0.05). A lower prevalence of dietary supplements for the overall strengthening of the body among those who assessed their health negatively was observed in 2023 (*p* < 0.05). No association between the consumption of dietary supplements for other purposes and the subjective assessment of personal health was observed (*p* > 0.05) (Table 7).

The distribution of the respondents by the consumption of exact supplements was similar in 2022 and 2023. This was observed among the whole sample of consumers and in a subgroup of those who assessed their health negatively. The most prevalent dietary supplement was vitamin D. Moreover, highly prevalent were the complexes of vitamins and minerals: magnesium, vitamin C, omega-3 fatty acids, vitamins of the B group, and fish oil. Other dietary supplements were two or more times less prevalent (Table 8).

In 2022, among the respondents who assessed their health negatively, the consumption of B-group vitamins, other vitamins (other than those listed in Table 7), magnesium and potassium was higher than among those who assessed their health positively (*p* < 0.05). In 2022, the consumption of selenium was lower among those who assessed their health negatively (*p* < 0.05). In 2023, no association between the subjective assessment of personal health and the consumption of exact dietary supplements was observed (*p* > 0.05). Also, the distribution of the respondents who assessed their health negatively by the consumption of exact supplements was similar to that observed in 2022 (*p* > 0.05) (Table 8).

## 4. Discussion

This study revealed the change in the prevalence of the consumption of dietary supplements among the working-age residents of Lithuania in the period from 2021 to 2023, covering the end of the COVID-19 pandemic and two years later, including the period of the wide-scale war in Ukraine. The results of this study revealed changes in the consumption of dietary supplements in many social and demographic groups, as well as in a subgroup of those who negatively assessed their personal health. Taking into account such a period of time, to our knowledge, up to this date, this study is the first published country-representative study of this topic.

Our results revealed that the prevalence of the consumption of dietary supplements was significantly lower in 2022, after the COVID-19 pandemic, when a wide-scale war in Ukraine began. Results of the 2023 survey showed the stabilization of the prevalence of the consumption of dietary supplements and even a higher prevalence of consumption among females, younger residents of Lithuania, and those from families with at least two members. The post-pandemic decrease in the prevalence of the consumption of dietary supplements was expected in advance because a study conducted in Poland showed that the prevalence of consumption was likely to decrease during the third wave of the pandemic [9]. Despite the decrease, the prevalence of consumption in 2022 and 2023 was higher than in 2017 and 2019 [8,14].

Despite the lower prevalence of consumption of dietary supplements in 2022, our study revealed that there were several social and demographic groups where no significant changes in the prevalence of consumption of dietary supplements were observed. These included respondents from one-person families, those with children under 18 years old, and those who indicated health strengthening as the main criteria for the selection of foods. Other researchers emphasize additional factors, such as the Russian–Ukrainian war, that could impact nutrition in the post-pandemic period, which caused a socioeconomic crisis [15]. Socioeconomic factors, as shown in other studies, are important determinants for the consumption of dietary supplements [2].

Contrary to the findings of other researchers, our study revealed no significant difference in the prevalence of consumption of dietary supplements between those with positive and negative assessments of personal health [7]. The higher prevalence of the consumption of dietary supplements was observed only during the pandemic, in 2021, but not in the post-pandemic period. What is more, our study revealed opposite tendencies in the prevalence of consumption of dietary supplements among those with negative assessments of their personal health. While in the general population the prevalence was higher among females, those with university education, those from larger municipalities, employed residents, those with higher income, and those selecting foods with the aim of health strengthening, among the residents with negative assessment of their personal health, a higher prevalence in the consumption of dietary supplements was observed among older adults, those with non-university education, unemployed residents, those with lower income, and those selecting foods according to other than health strengthening criteria. These results seem to be contradictory to the findings of other researchers, who presented that lower socio-economic status is associated with more frequent inadequacy of dietary supplements [16]. Unfortunately, we were not able to assess the actual nutrition of the participants of our study; subsequently, we were not able to assess the adequacy of an intake of dietary supplements. Notably, the improvement of health [17,18,19], advertising [20], and other factors [2] are presented as important determinants for the consumption of dietary supplements by other researchers. However, concerns about the inadequate consumption of dietary supplements have been raised for quite some time [21].

Similar to our results, other studies also show a high prevalence of consumption of dietary supplements containing vitamin D, multivitamins, vitamin C, omega-3 fatty acids, probiotics, and zinc [22,23]. In our study, the prevalence of these dietary supplements was observed in the general samples also in the subgroups of respondents with a negative assessment of personal health. However, these subgroups presented several differences. Despite a few other mismatches, different from the general population, those with a negative assessment of their personal health more frequently selected dietary supplements for better digestion, regulation of sleep and boosting the nervous system. Despite the fact that only in 2021, a significantly higher prevalence of the consumption of dietary supplements for strengthening the cardiovascular system was observed among those with a negative assessment of their personal health, in 2022, such group of respondents more frequently consumed magnesium and potassium, as well as the B-group vitamins. These findings possibly show the necessity to adapt or specifically target nutrition education and health promotion interventions according to the assessment of personal health [24].

### Limitations

We were able to assess only the subjective views of the respondents on the consumption of dietary supplements because of the cross-sectional design of the study. A longitudinal study would have allowed us to objectively assess the changes in consumption of dietary supplements at an individual level. In addition, this would have allowed us to use more advanced statistical methods to predict the change in the consumption of dietary supplements. On the other hand, this study included three country-representative samples, which were larger than sufficient to assess the consumption of dietary supplements among adult residents of Lithuania.

Also, in order to simplify the presentation and interpretation of the results, we converted the age-representing variable into binary. Despite the fact that it revealed some significant differences in the consumption of dietary supplements, in the upcoming studies, it would be beneficial to perform a more detailed analysis because such conversion might hide some subgroups that might significantly differ from each other.

Despite the fact that we analyzed the consumption of dietary supplements with respect to many social, demographic and health-related factors, there might be important factors that were not included in our analysis.

## 5. Conclusions

In comparison to 2021, among working-age Lithuanian residents, the prevalence of the consumption of dietary supplements was significantly lower in 2022, when the COVID-19 pandemic ended and a wide-scale war in Ukraine began, but in 2023 it was higher again. The lower prevalence was observed in most of the social and demographic groups, while a higher prevalence was observed among females, younger residents of Lithuania, and those from families with at least two members.

A negative assessment of personal health is associated with opposite tendencies in the prevalence of the consumption of dietary supplements in the general population. In terms of the general population, females, those with university education, those from larger municipalities, employed residents, those with higher income, and those selecting foods with the aim of health strengthening show a higher prevalence in the consumption of dietary supplements. Among the residents with negative assessments of their personal health, a higher prevalence in the consumption of dietary supplements is observed among older adults, those with non-university education, unemployed residents, those with lower income, and those selecting foods according to other than health strengthening criteria. Both, in the general population of the working-aged residents of Lithuania and among the residents with negative assessments of their personal health, residents without children under 18 years old, and those who suffered from a severe form of COVID-19 more frequently indicated the consumption of dietary supplements, while it did not differ with respect to the different marital status and number of family members.

The consumption prevalence of specific dietary supplements in the majority of cases does not differ between those with a negative or positive assessment of personal health. However, dietary supplements for strengthening the cardiovascular system, boosting the nervous system, regulating sleep, and improving digestion are more prevalent among those with a negative health assessment. This should be taken into account while preparing nutrition education and health promotion interventions.

## Figures and Tables

**Table 1:** Questions about the consumption of dietary supplements included in this study.

Question	Categories with Relevant Response Options *
Do you consume dietary supplements (vitamins, minerals, polyunsaturated fatty acids, plant-based preparations, etc.)?	Yes (yes, always/yes, more than 6 months per year/yes, 4–6 months per year/yes, 2–3 months per year/yes, 1 month per year/yes, but shortly or accidentally) No (no, I do not consume) Excluded from the analysis (I do not know/cannot answer)
What dietary supplements and what for have you taken over the last 12 months? **	For strengthening the immune system For disease prevention and the overall strengthening of the body/For energy boosting/For eye care/For boosting memory/For boosting the nervous system/For strengthening the cardiovascular system/For strengthening the joints, bones/For better digestion For sleep regulation/For athletes/For weight regulation/For protection against the COVID-19 infection/Other
What is the most important for you when selecting food products?	Health strengthening (Benefits to health) Other (Taste/Price/Preferences of other family members/The necessity of diet/Other) Excluded from the analysis (I do not know/cannot answer)
Which of the dietary supplements have you consumed/taken over the last 12 months? (the question used only in the 2022 and 2023 surveys) **	Complex of vitamins and minerals/Complex of vitamins/Complex of minerals/Omega-3 fatty acids/Fish oil/Plant-based/Targeted at the immune system/Targeted at the cardiovascular system/Targeted at the nervous system/Targeted at the general strengthening of the body/Vitamin C/Vitamin D/Vitamin A/Vitamins of the B group/Folic acid/Other vitamins/Iron/Magnesium/Potassium/Calcium/Zinc/Selenium/Other minerals/Coenzyme Q10/Probiotics/Other/I do not know
How would you assess your health?	Positively (Very good/Rather good/Neither good nor bad) Negatively (Rather bad/Very bad) Excluded from the analysis (I do not know/cannot answer)
*** Please select the appropriate statements for you: (Level of exposure to COVID-19) **	I am suffering (or suffered) from COVID-19/There is (or was) a member in my family who is suffering (or suffered) from COVID-19/My friends, acquaintances, neighbors are suffering (or suffered) from COVID-19 in their families/I do not know anyone who is suffering (or suffered) from COVID-19
Please select the most appropriate statement about your COVID-19 infection:	Suffered from the asymptomatic or mild form of COVID-19 (I had an asymptomatic form of this disease/I had a mild form of this disease)/Suffered from a severe COVID-19 form (I had a severe form of this disease/I had a very severe form of this disease)

* In case of larger categories, the response options are provided in brackets; ** Selection of multiple answer options available; ***—in 2023, the respondents were asked to indicate the exposure to COVID-19 over the last 12 months while in the previous surveys such period of time was not defined.

**Table 2:** Distribution of the respondents by social and demographic factors.

Factor	Sample of 2021		Sample of 2022		Sample of 2023	
	N	Relative Frequency (%)	N	Relative Frequency (%)	N	Relative Frequency (%)
Consumption of dietary supplements	1587		1579		1573	
Yes	1240	78.1	1131	71.6	1163	72.7
No	347	21.9	448	28.4	410	27.3
Sex	1600		1600		1600	
Male	792	49.5	800	50.0	785	49.0
Female	808	50.5	800	50.0	815	51.0
Age	1600		1600		1600	
41 years old or younger	769	48.1	784	49.0	753	47.1
42 years old or older	831	51.9	816	51.0	847	52.9
Education	1484		1495		1506	
Non-university education	474	31.9	495	33.1	465	30.9
University education	1010	68.1	1000	66.9	1041	69.1
Place of residence	1600		1600		1600	
City	678	42.4	686	42.9	670	41.9
A small town or village	922	57.6	914	57.1	930	58.1
Marital status *	1600		1600		1600	
Single	636	39.8	644	40.2	508	31.8
Married	964	60.2	956	59.8	1092 ^	68.2 ^
Number of family members	1600		1600		1600	
Two or more	1408	88.0	1370	85.6	1369	85.6
One	192	12.0	230 ^	14.4 ^	231	14.4
With children under 18 years old	1600		1600		1600	
No	991	61.9	967	60.4	990	61.9
Yes	609	38.1	633	39.6	610	38.1
Employment	1494		1471		1480	
Employed	1174	78.6	1135	77.1	1139	76.9
Unemployed	321	21.4	336	22.9	341	23.1
Income	1248		1226		1275	
Lower	412	33.0	437	35.7	409	32.1
Higher	836	67.0	789	64.3	866	67.9
Food selection criteria *	1569		1541		1576	
Health strengthening	489	31.1	286	18.5	381 ^	24.2 ^
Other	1081	68.9	1256 ^	81.5 ^	1195	75.8
COVID-19 among family members	1600		1600		1600	
There were no COVID-19 cases in the respondent’s family	970	60.6	393	24.6	1031	64.5
The respondent or his/her family members suffered from COVID-19	630	39.4	1207	75.4	569	35.5
Severeness of COVID-19	343		385		385	
Suffered from the asymptomatic or mild form of COVID-19	264	77.1	293	76.2	287	74.4
Suffered from a severe form of COVID-19	78	22.9	92	23.8	99	25.6
Subjective assessment of health status	1587		1569		1570	
Negative	115	7.3	136	8.7	151	9.6
Positive	1472	92.7	1433	91.3	1419	90.4

* a significant difference (*p* < 0.05) between all the three samples (2021, 2022 and 2023); ^ a significantly higher prevalence (*p* < 0.05) compared to the sample collected one year before (2021 vs. 2022, 2022 vs. 2023).

**Table 3:** Distribution of the respondents by purpose for the consumption of food supplements and the consumption within the past 12 months in the three samples.

The Purpose for the Consumption of Dietary Supplements	Sample of 2021		Sample of 2022		Sample of 2023		*p*-Value
	N	Relative Frequency, %	N	Relative Frequency, %	N	Relative Frequency, %	
Strengthening the immune system	607	49.1	546	48.5	530	45.6	0.187
The overall strengthening of the body	539	43.6	453	40.3	483	41.5	0.257
Energy boosting	171	13.8	170	15.1	166	14.3	0.667
Eye care	163	13.2	165	14.7	173	14.9	0.428
Boosting memory	134	10.8	140	12.5	128	11.0	0.409
Boosting the nervous system	291	23.5	262	23.3	289	24.8	0.637
Strengthening the cardiovascular system	333	26.9	279	24.8	314	27	0.398
Strengthening the joints, bones	310	25.1	260	23.1	301	25.9	0.289
Better digestion	202	16.3	167	14.8	180	15.5	0.606
Regulation of sleep	120	9.7	133	11.8	147	12.6	0.063
For athletics	69	5.6	49	4.4	49	4.2	0.222
Reduction/control of body weight	93	7.5	68	6.0	73	6.3	0.297
Protection against the COVID-19 infection *	63	5.1	47	4.2	24 ^@^	2.1 ^@^	<0.001
Other	86	6.9	84	7.5	90	7.7	0.755
Total	1237		1124		1163		

* a significant difference (*p* < 0.05) between all the three samples (2021, 2022 and 2023); ^@^ a significantly lower prevalence (*p* < 0.05) compared to the sample collected one year before (2021 vs. 2022, 2022 vs. 2023).

**Table 4:** Distribution of the respondents who indicated the consumption of dietary supplements by social and demographic factors in the three samples.

Factor	Sample of 2021		Sample of 2022		Sample of 2023		*p*-Value
	N	%	N	%	N	%	
Sex	1237		1124		1162		
Male *	590 ^b^	47.7 ^b^	529 ^b@^	47.1 ^b@^	521 ^b^	44.8 ^b^	0.002
Female *	647 ^a^	52.3 ^a^	595 ^a@^	52.9 ^a@^	641 ^a^^	55.2 ^a^^	0.018
Age	1237		1124		1163		
41 years old or younger *	580	46.9	530 ^b@^	47.2 ^b@^	560 ^^a^	48.2 ^^a^	0.002
42 years old or older *	657	53.1	594 ^a@^	52.8 ^a@^	603 ^b^	51.8 ^b^	0.001
Education	1166		1065		1101		
Non-university education *	350 ^b^	30.0 ^b^	309 ^b@^	29.0 ^b@^	306 ^b^	27.8 ^b^	0.002
University education *	816 ^a^	70.0 ^a^	756 ^a@^	71.0 ^a@^	795 ^a^	72.2 ^a^	0.013
Place of residence	1238		1124		1163		
City *	546 ^a^	44.1 ^a^	507 ^a@^	45.1 ^a@^	515 ^a^	44.3 ^a^	0.025
A small town or village *	692 ^b^	55.9 ^b^	617 ^b@^	54.9 b^@^	648 ^b^	55.7 ^b^	0.005
Marital status	1238		1124		1163		
Single *	500	40.4	449 ^@^	39.9 ^@^	361	31.0	0.004
Married *	738	59.6	675 ^@^	60.1 ^@^	802	69.0	0.020
Number of family members	1237		1124		1163		
Two or more *	1080	87.3	950 ^@^	84.5 ^@^	1002 ^	86.2 ^	<0.001
One *	157	12.7	174	15.5	161	13.8	0.024
With children under 18 years old	1237		1124		1162		
No *	782 ^a^	63.2 ^a^	685 ^@^	60.9 ^@^	710	61.1	<0.001
Yes	455 ^b^	36.8 ^b^	439	39.1	452	38.9	0.209
Employment	1162		1046		1082		
Employed *	935 ^a^	80.5 ^a^	824 ^a@^	78.8 ^a@^	841	77.7	0.001
Unemployed	227 ^b^	19.5 ^b^	222 ^b^	21.2 ^b^	241	22.3	0.311
Income	975		861		930		
Lower	306 ^b^	31.4 ^b^	289 ^b@^	33.6 ^b@^	285	30.6	0.095
Higher *	669 ^a^	68.6 ^a^	572 ^a@^	66.4 ^a@^	645	69.4	0.002
Food selection criteria	1221		1102		1152		
Health strengthening	399 ^a^	32.7 ^a^	221 ^a^	20.1 ^a^	311 ^a^	27.0 ^a^	0.181
Other *	822 ^b^	67.3 ^b^	881 ^b@^	79.9 ^b@^	841 ^b^	73.0 ^b^	0.009
COVID-19 among family members	1240		1131		1163		
There were no COVID-19 cases in the respondent’s family *	738	59.5	259 ^b@^	22.9 ^b@^	726 ^b^	62.4 ^b^	0.001
The respondent or his/her family members suffered from COVID-19 *	502	40.5	872 ^a@^	77.1 ^a@^	437 ^a^^	37.6 ^a^^	<0.001
Severeness of COVID-19	267		276		301		
Suffered from the asymptomatic or mild form of COVID-19	200	74.9	214	77.5	216 ^b^	71.8 ^b^	0.720
Suffered from a severe form of COVID-19 *	67	25.1	62 ^@^	22.5 ^@^	85 ^a^^	28.2 ^a^^	0.003
Subjective assessment of health status	1237		1124		1163		
Negative *	100 ^a^	8.1 ^a^	98 ^@^	8.7 ^@^	117	10.1	0.021
Positive *	1137 ^b^	91.9 ^b^	1026 ^@^	91.3 ^@^	1046	89.9	0.003

* a significant difference (*p* < 0.05) between all the three samples (2021, 2022 and 2023); ^ a significantly higher prevalence (*p* < 0.05) compared to the sample collected one year before (2021 vs. 2022, 2022 vs. 2023); ^@^ a significantly lower prevalence (*p* < 0.05) compared to the sample collected one year before (2021 vs. 2022, 2022 vs. 2023); ^a^—a significantly higher prevalence within the sample (*p* < 0.05); ^b^—a significantly lower prevalence within the sample (*p* < 0.05).

**Table 5:** Distribution of the respondents who assessed their health negatively and social and demographic factors in the three samples.

Factor	Sample of 2021		Sample of 2022		Sample of 2023		*p*-Value
	N	%	N	%	N	%	
Consumption of dietary supplements	115		135		150		
Yes	100	87.1	98	72.9	117	78.3	
No	15	12.9	37	27.1	32	21.7	
Sex	115		137		151		
Male	60	52.2	74	54.0	59 ^b^	39.1 ^b^	0.322
Female *	55	47.8	63	46.0	92 ^a^^	60.9 ^a^^	0.003
Age	115		137		151		
41 years old or younger	40 ^b^	34.8 ^b^	52 ^b^	38.0 ^b^	56 ^b^	37.1 ^b^	0.167
42 years old or older	75 ^a^	65.2 ^a^	85 ^a^	62.0 ^a^	95 ^a^	62.9 ^a^	0.305
Education	109		130		144		
Non-university education	47 ^a^	43.1 ^a^	48	36.9	59 ^a^	41.0 ^a^	0.268
University education	62 ^b^	56.9 ^b^	82	63.1	85 ^b^	59.0 ^b^	0.125
Place of residence	115		136		151		
City	53	46.1	53	39.0	53	35.1	0.991
A small town or village *	62	53.9	83	61.0	98	64.9	0.011
Marital status	115		137		151		
Single	48	41.7	60	43.8	58 ^a^	38.4 ^a^	0.063
Married	67	58.3	77	56.2	93 ^b^	61.6 ^b^	0.379
Number of family members	115		136		151		
Two or more	98	85.2	116	85.3	126	83.4	0.073
One	17	14.8	20	14.7	25	16.6	0.658
With children under 18 years old	115		136		151		
No	82 ^a^	71.3 ^a^	93	68.4	110 ^a^	72.8 ^a^	0.089
Yes	33 ^b^	28.7 ^b^	43	31.6	41 ^b^	27.2 ^b^	0.475
Employment	110		132		138		
Employed	72 ^b^	65.5 ^b^	95 ^	72.0 ^	95 ^b^	68.8 ^b^	0.056
Unemployed	38 ^a^	34.5 ^a^	37	28.0	43 ^a^	31.2 ^a^	0.805
Income	85		114		129		
Lower *	37 ^a^	43.5 ^a^	57 ^a^^	50 ^a^^	58 ^a^	45.0 ^a^	0.043
Higher	48 ^b^	56.5 ^b^	57 ^b^	50.0 ^b^	71 ^b^	55.0 ^b^	0.125
Food selection criteria	113		131		150		
Health strengthening	25 ^b^	22.1 ^b^	13 ^b^	9.9 ^b^	28	18.7	0.212
Other	88 ^a^	77.9 ^a^	118 ^a^	90.1 ^a^	122	81.3	0.195
COVID-19 among family members	115		136		151		
There were no COVID-19 cases in the respondent’s family	67	58.3	33	24.3	92	60.9	0.193
The respondent or his/her family members suffered from COVID-19	48	41.7	103	75.7	59	39.1	0.228
Severeness of COVID-19	71		21		41		
Suffered from the asymptomatic or mild form of COVID-19 *	60	84.5	13 ^@^	61.9 ^@^	25 ^b^^	61.0 b^	<0.001
Suffered from a severe form of COVID-19	11	15.5	8	38.1	16 ^a^	39.0 ^a^	0.308

* a significant difference (*p* < 0.05) between all the three samples (2021, 2022 and 2023); ^ a significantly higher prevalence (*p* < 0.05) compared to the sample collected one year before (2021 vs. 2022, 2022 vs. 2023); ^@^ a significantly lower prevalence (*p* < 0.05) compared to the sample collected one year before (2021 vs. 2022, 2022 vs. 2023); ^a^—a significantly higher prevalence within the sample (*p* < 0.05); ^b^—a significantly lower prevalence within the sample (*p* < 0.05).

**Table 6:** Distribution of the respondents who indicated the consumption of dietary supplements and negatively assessed their health by social and demographic factors in the three samples.

Factor	Sample of 2021		Sample of 2022		Sample of 2023		*p*-Value
	N	%	N	%	N	%	
Sex	100		98		117		
Male *	52	52.0	50 ^@^	51.0 ^@^	42 ^b^	35.9 ^b^	0.032
Female	48	48.0	48	49.0	75 ^a^	64.1 ^a^	0.303
Age	100		98		117		
41 years old or younger	33 ^b^	33.0 ^b^	35 ^b^	35.7 ^b^	40 ^b^	34.2 ^b^	0.252
42 years old or older *	67 ^a^	67.0 ^a^	63 ^a@^	64.3 ^a@^	77 ^a^	65.8 ^a^	0.049
Education	95		93		110		
Non-university education *	40 ^a^	42.1 ^a^	27 ^@^	29.0 ^@^	43 ^a^	39.1 ^a^	0.007
University education	55 ^b^	57.9 ^b^	66	71.0	67 ^b^	60.9 ^b^	0.271
Place of residence	100		98		117		
City	47	46.5	39 ^@^	39.8 ^@^	44	37.6	0.127
A small town or village	53	53.5	59 ^@^	60.2 ^@^	73	62.4	0.065
Marital status	100		98		117		
Single	41	40.6	41 ^@^	41.8 ^@^	43	36.8	0.119
Married	59	59.4	57 ^@^	58.2 ^@^	74	63.2	0.060
Number of family members	100		98		117		
Two or more *	84	84.0	81 ^@^	82.7 ^@^	96	82.2	0.023
One	16	16.0	17	17.3	21	17.8	0.595
With children under 18 years old	100		98		117		
No *	73 ^a^	73.0 ^a^	68 ^@^	69.4 ^@^	87 ^a^	74.4 ^a^	0.030
Yes	27 ^b^	27.0 ^b^	30	30.6	30 ^b^	25.6 ^b^	0.480
Employment	96		94		106		
Employed *	65 ^b^	67.7 ^b^	70 ^@^	74.5 ^@^	73 ^b^	68.9 ^b^	0.024
Unemployed	31 ^a^	32.3 ^a^	24	25.5	33 ^a^	31.1 ^a^	0.231
Income	73		79		100		
Lower	31 ^a^	42.5 ^a^	38 ^a^	48.1 ^a^	41 ^a^	41.0 ^a^	0.181
Higher	42 ^b^	57.5 ^b^	41 ^b^	51.9 ^b^	59 ^b^	59.0 ^b^	0.105
Food selection criteria	98		97		117		
Health strengthening *	22 ^b^	22.4 ^b^	10 ^b^	10.3 ^b^	28 ^	23.9 ^	0.047
Other *	76 ^a^	77.6 ^a^	87 ^a@^	89.7 ^a@^	89	76.1	0.046
COVID-19 among family members	100		98		117		
There were no COVID-19 cases in the respondent’s family	55	55.0	18	18.4	71	60.7	0.224
The respondent or his/her family members suffered from COVID-19 *	45	45.0	80	81.6	46	39.3	0.665
Severeness of COVID-19	22		13		32		
Suffered from the asymptomatic or mild form of COVID-19 *	6 ^b^	27.3 ^b^	10	76.9	18 ^b^	56.3 ^b^	0.047
Suffered from a severe form of COVID-19 *	16 ^a^	72.7 ^a^	3 ^@^	23.1 ^@^	14 ^a^^	43.8 ^a^^	0.010

* a significant difference (*p* < 0.05) between all the three samples (2021, 2022 and 2023); ^ a significantly higher prevalence (*p* < 0.05) compared to the sample collected one year before (2021 vs. 2022, 2022 vs. 2023); ^@^ a significantly lower prevalence (*p* < 0.05) compared to the sample collected one year before (2021 vs. 2022, 2022 vs. 2023); ^a^—a significantly higher prevalence within the sample (*p* < 0.05); ^b^—a significantly lower prevalence within the sample (*p* < 0.05).

**Table 7:** Distribution of the respondents who negatively assessed their health by purpose for the consumption of food supplements in the three samples.

The Purpose for the Consumption of Dietary Supplements	Sample of 2021		Sample of 2022		Sample of 2023		*p*-Value *
	N	Relative Frequency, %	N	Relative Frequency, %	N	Relative Frequency, %	
Strengthening the immune system	47	47.0	46	46.9	54	46.2	0.990
The overall strengthening of the body	37	37.0	32	32.7	36 ^b^	30.8 ^b^	0.615
Energy boosting	15	14.9	17	17.3	20	17.1	0.869
Eye care	11	11.0	14	14.3	16	13.6	0.767
Boosting memory	10	9.9	15	15.3	17	14.5	0.470
Boosting the nervous system	32 ^a^	31.7 ^a^	33 ^a^	33.3 ^a^	31	26.3	0.489
Strengthening the cardiovascular system *	44 ^a^	44.0 ^a^	26 ^@^	26.5 ^@^	38	32.5	0.031
Strengthening the joints, bones	31	31.0	28	28.6	28	23.9	0.494
Better digestion	26 ^a^	26.0 ^a^	27 ^a^	27.6 ^a^	28 ^a^	23.9 ^a^	0.830
Regulation of sleep	18 ^a^	18.0 ^a^	18 ^a^	18.4 ^a^	21	17.9	0.996
For athletics	3	3.0	3	3.1	2	1.7	0.775
Reduction/control of body weight	3	3.0	8	8.1	8	6.8	0.287
Protection against the COVID-19 infection	6	6.0	4	4.1	2	1.7	0.254
Total	100		98		117		

* a significant difference (*p* < 0.05) between all the three samples (2021, 2022 and 2023); ^@^ a significantly lower prevalence (*p* < 0.05) compared to the sample collected one year before (2021 vs. 2022, 2022 vs. 2023); ^a^—a significantly higher prevalence within the sample (*p* < 0.05); ^b^—a significantly lower prevalence within the sample (*p* < 0.05).

**Table 8:** Distribution of the respondents by the consumption of exact dietary supplements in the samples of 2022 and 2023, with respect to a subjective health assessment.

Dietary Supplements	Whole Sample of 2022		Whole Sample of 2023 (N = 1174)		*p*-Value *	Among Those Who Assessed Their Health Negatively in 2022		Among Those Who Assessed Their Health Negatively in 2023		*p*-Value *
	N	Relative Frequency (%)	N	Relative Frequency (%)		N	Relative Frequency (%)	N	Relative Frequency (%)	
Complex of vitamins and minerals	396	35.2	430	37.0	0.377	32	32.3	43	36.8	0.496
Complex of vitamins	89	7.9	75	6.4	0.175	10	10.2	10	8.5	0.695
Complex of minerals	31	2.8	37	3.2	0.549	5	5.1	2	1.7	0.167
Omega-3 fatty acids	332	29.5	360	31.0	0.452	31	31.6	35	29.9	0.824
Fish oil	282	25.1	304	26.1	0.557	24	24.2	24	20.5	0.511
Plant-based	91	8.1	96	8.3	0.885	7	7.1	9	7.7	0.862
Targeted at the immune system	154	13.7	139	12.0	0.214	11	11.1	10	8.5	0.526
Targeted at the cardiovascular system	115	10.2	126	10.8	0.634	15	15.3	17	14.5	0.898
Targeted at the nervous system	110	9.8	94	8.1	0.155	14	14.3	12	10.3	0.382
Targeted at the general strengthening of the body	109	9.7	118	10.1	0.715	9	9.2	12	10.2	0.773
Vitamin C	354	31.5	340	29.3	0.246	28	28.3	26	22.2	0.305
Vitamin D	539	47.9	534	45.9	0.339	54	54.5	60	51.3	0.632
Vitamin A	67	6.0	76	6.5	0.567	5	5.1	9	7.7	0.432
B Group vitamins	288	25.6	307	26.4	0.664	34 ^a^	34.7 ^a^	32	27.4	0.266
Folic acid	70	6.2	80	6.9	0.526	5	5.1	9	7.6	0.432
Other vitamins	25	2.2	36	3.1	0.195	5 ^a^	5.1 ^a^	1	0.9	0.062
Iron	151	13.4	167	14.4	0.517	12	12.2	13	11.1	0.817
Magnesium	380	33.8	436	37.5	0.064	45 ^a^	45.9 ^a^	49	41.5	0.598
Potassium	161	14.3	188	16.2	0.218	21 ^a^	21.4 ^a^	24	20.5	0.900
Calcium	127	11.3	129	11.1	0.881	12	12.2	10	8.5	0.387
Zinc	181	16.1	163	14.0	0.165	13	13.3	14	12	0.796
Selenium	104	9.3	101	8.7	0.639	3 ^b^	3.1 ^b^	9	7.7	0.136
Other minerals	19	1.7	26	2.2	0.346	2	2.0	4	3.4	0.533
Coenzyme Q10	58	5.2	55	4.7	0.638	1	1.0	7	6.0	0.054
Probiotics	106	9.4	120	10.3	0.473	13	13.3	17	14.5	0.767
Other	38	3.4	48	4.1	0.346	2	2.0	4	3.4	0.533
Did not know/Could not answer	10	0.9	11	0.9	0.886	1	1.0	1	0.9	0.905
Total	1124		1163			98		117		

*—*p*-values were calculated comparing the frequencies between the samples collected in 2022 and 2023; ^a^—a significantly higher prevalence within the sample (*p* < 0.05); ^b^—a significantly lower prevalence within the sample (*p* < 0.05).

## Data Availability

The raw data supporting the conclusions of this article will be made available by the authors on request.
